# Uvular Necrosis: Day-to-Day Progression of a Rare Postoperative Complication

**DOI:** 10.7759/cureus.45132

**Published:** 2023-09-12

**Authors:** Michael A De Freitas, Laura Santiago Caobi, Oswaldo A Guevara Tirado, Luis A Borroto, Pedro Whatts

**Affiliations:** 1 Anesthesiology, Ponce Health Sciences University, Ponce, PRI; 2 Pediatrics, Ponce Health Sciences University, Ponce, PRI; 3 Anesthesiology, St. Luke's Episcopal Hospital, Ponce, PRI

**Keywords:** uvular necrosis, uvular, airway, sedation, endotracheal, intubation, anesthesia, pain, throat discomfort, necrosis

## Abstract

Uvular necrosis is a rare post-procedural complication thought to be caused by mechanical compression of the uvula during endotracheal intubation. We described the day-to-day progression of uvular necrosis after right shoulder acromioclavicular (AC) joint reconstruction. We present a case of a 22-year-old male who visited the emergency department after sustaining a right shoulder trauma. Diagnosis of a type V AC dislocation with total coracoclavicular ligament tear was established. On day one, after endotracheal intubation for the right shoulder AC joint reconstruction, the patient complained of severe throat pain that progressed to odynophagia, dysphagia, and choking. Examination revealed an erythematous uvula with well-demarcated necrotic tissue. He was managed conservatively with acetaminophen and ice chips. Day-to-day symptom progression description may guide physicians in managing postoperative uvular necrosis.

## Introduction

Uvular necrosis is an uncommon postoperative complication with an incidence rate of 0.03% [1]. It presents with various symptoms ranging from persistent throat discomfort to difficulty swallowing and upper airway obstruction [2]. The etiology of uvular necrosis remains unclear. However, it is believed to be caused by mechanical compression of the vessels that supply blood to the uvula by the placement of oropharyngeal devices [3]. Although reports exist of uvular necrosis following upper gastrointestinal endoscopy and bronchoscopy [4-6], it is more frequently associated with the use of endotracheal tubes and laryngeal masks [3,6]. Risk factors include being male due to their neck anatomy [6] and having an enlarged uvula [2,7]. There is limited information about uvular necrosis after shoulder surgery in the literature, partly because of the infrequent nature of this postoperative complication [8]. Considering the enormous discomfort and severe pain associated with uvular necrosis, it is crucial to minimize risks to avoid the development of this complication. Our case report aims to describe a rare case of uvular necrosis following endotracheal intubation during right shoulder surgery and to provide more information about this unusual postoperative complication to the scientific community and the general population.

## Case presentation

A 22-year-old male presented to the emergency department due to a direct traumatic right shoulder injury following a snowboarding accident. The right shoulder was stabilized using a sling, and acetaminophen was given as needed for pain management. Following radiographic imaging and evaluation by orthopedic surgery, the patient was diagnosed with a right type V acromioclavicular (AC) dislocation with a total coracoclavicular ligament tear. Right shoulder AC joint reconstruction using allograft was indicated. The patient’s medical history was significant for essential hypertension well-controlled on candesartan. He reported mild alcohol consumption (one to two drinks per week) and occasional tetrahydrocannabinol (THC) use (less than two times per year); no history of tobacco use was reported. The patient's body mass index (BMI) was 26.9 lbs/in^2^.

**Figure 1:**
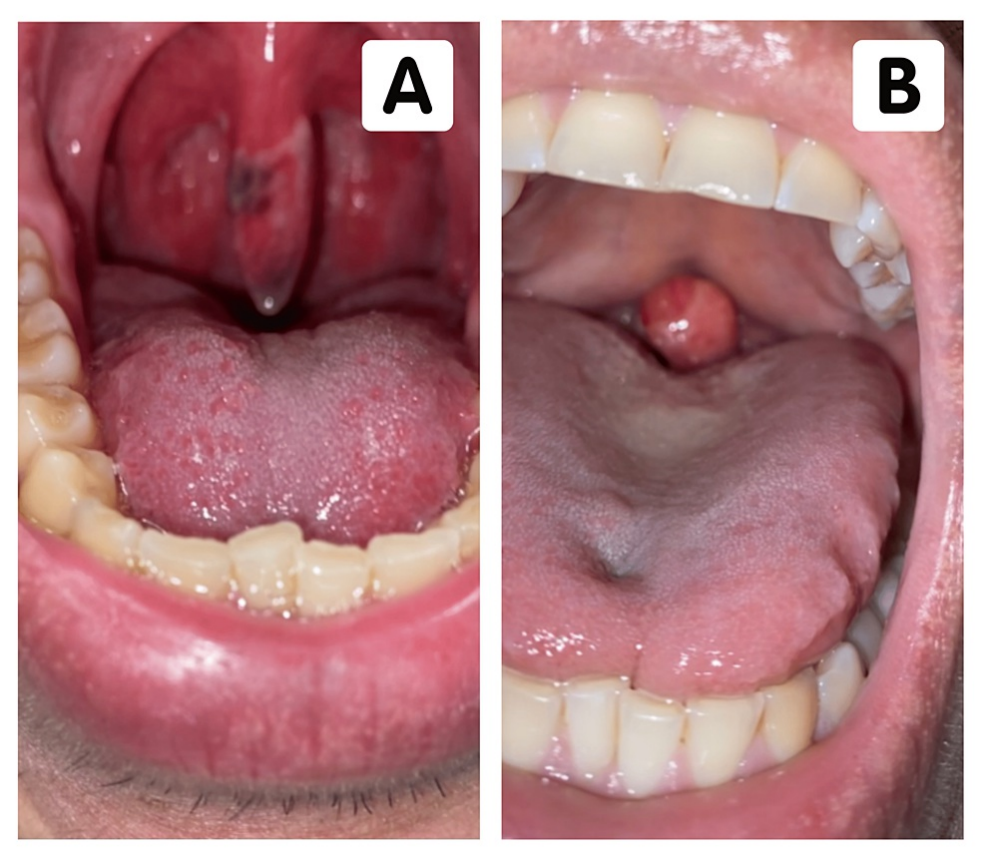
(A) The oropharynx of the patient 24 hours post procedure with early signs of uvular necrosis. (B) The oropharynx of the patient in a supine position on postoperative day two revealed airway obstruction.

In the operating room, the patient was placed in the supine position and underwent an uneventful induction of general anesthesia. Direct laryngoscopy using a size 8.0 endotracheal tube was atraumatic and easily performed. Diagnostic arthroscopy revealed obliteration of the AC and coracoclavicular ligaments. The surgical procedure yielded a successful coracoclavicular joint reconstruction with an estimated blood loss of 10 mL. The patient was then extubated and transferred to the recovery room in stable condition. Following discharge, the patient complained of moderate throat pain with overnight progression to severe pain, odynophagia, and dysphagia. Examination of the oropharynx at 24 hours post procedure revealed an erythematous uvula with well-demarcated necrotic tissue along the right lateral aspect and tip (Figure 1A). Examination of the pharynx and regional lymph nodes was unremarkable, and a diagnosis of uvular necrosis was made.

**Figure 2:**
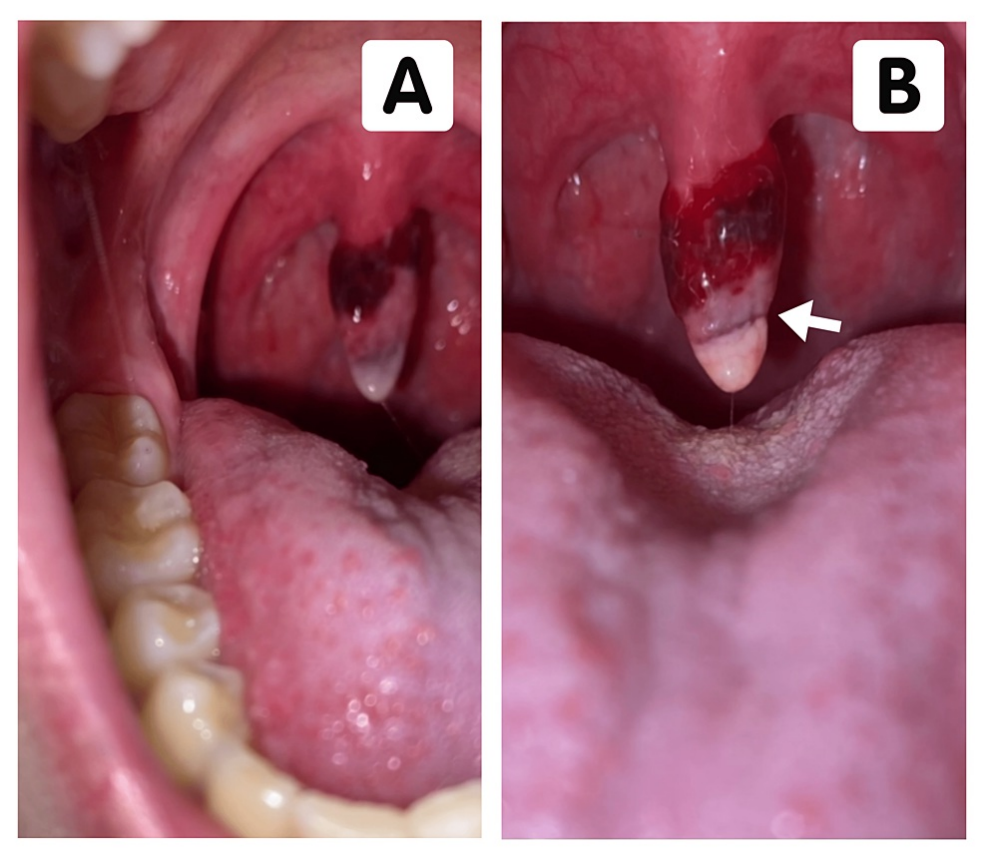
(A) The oropharynx of the patient on postoperative day two demonstrated circumferential necrotic expansion of the proximal one-third and tip. (B) The oropharynx of the patient on postoperative day three demonstrates necrosis and erythema >80% of the total uvular surface. The uvula exhibits an elongated whitish tip with a black border (arrow) differentiating healthy tissue from necrotic tissue.

On postoperative day two, the patient complained of unresolved symptoms and abrupt awakenings from sleep due to the sensation of choking. He described that when sleeping in the supine position, the necrotic uvula transiently obstructed his airway, prompting him to sleep in a semi-recumbent position (Figure 1B). That evening, the patient was evaluated by the otolaryngologist, and physical examination showed an elongated uvula with a circumferential necrotic expansion of the proximal one-third and tip (Figure 2A). The patient was reassured that his condition was self-limiting, and acetaminophen with ice chips was recommended for supportive treatment.

On postoperative day three, the patient reported oropharyngeal symptoms were at their peak in severity; albeit, the sensation of choking improved since the adjustment of the sleeping position. At this time, the patient was only able to tolerate a liquid diet consisting of juice and warm soup. Supportive management with acetaminophen and ice chips was still rigidly followed. Examination of the uvula showed necrosis and erythema involving nearly 80% of the total uvular surface area (Figure 2B). On postoperative day four, the patient reported decreased throat pain and discomfort and was tolerating a liquid diet with minimal pain.

**Figure 3:**
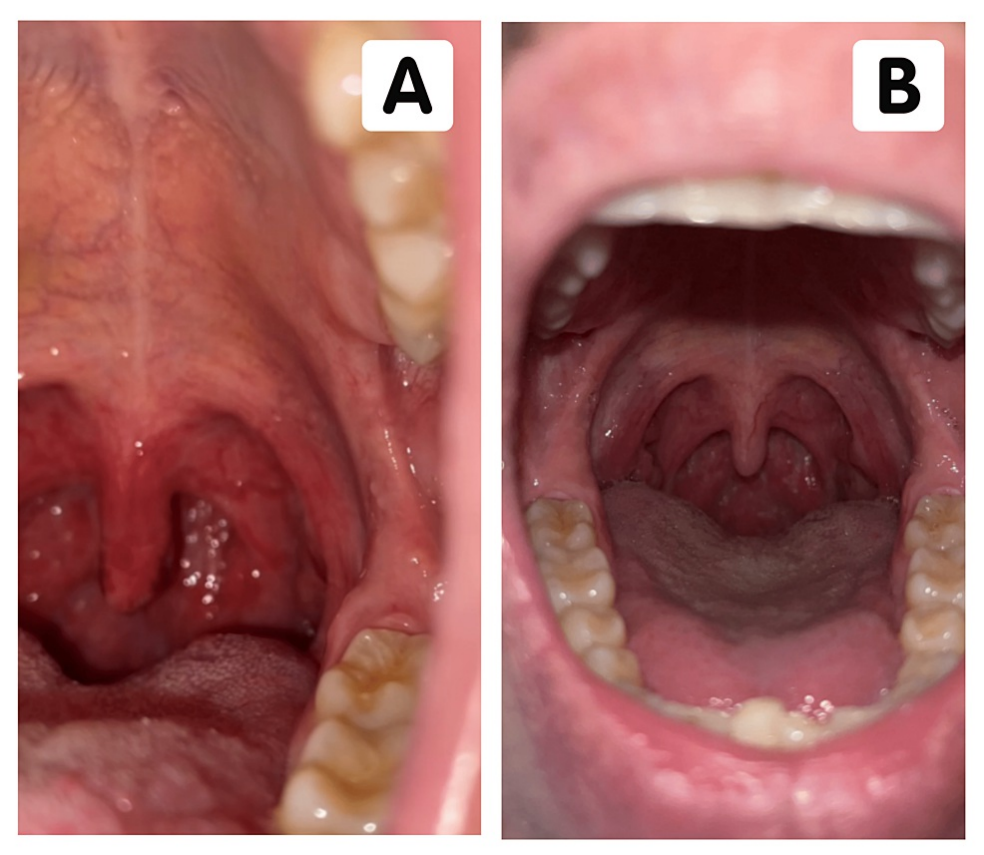
(A) The oropharynx of the patient on postoperative day five reveals improvement of necrotic changes. (B) The oropharynx of the patient one year later reveals no signs of apparent permanent changes in uvular tissue following postoperative uvular necrosis.

On postoperative day five, the uvula was mildly necrotic with resolving margins (Figure 3A). The patient reported markedly decreased throat pain with minimal odynophagia and dysphagia. He graduated to a mash-and-ground diet, which was tolerated adequately. In the following days, the patient reported complete resolution of symptoms with no residual disease and normal uvular appearance. At this point, supportive management was electively terminated by the patient with no incident. Evaluation one year later revealed no long-term sequelae of uvular necrosis with intact sensory function and no reported infection. The patient denied odynophagia, dysphagia, sleeping difficulties, hoarseness, or discomfort. Examination of the uvula showed no uvular deviation, baseline uvular length, and no signs of necrotic or fibrotic tissue (Figure 3B).

## Discussion

Pharyngeal irritation is a common cause of throat discomfort in patients following procedures that require the passage of instruments via the orotracheal or nasotracheal route [2,3,6-8]. In most cases, spontaneous improvement of symptoms occurs within one to two days with minimal requirement for analgesia [9,10]. Persistent throat discomfort accompanied by odynophagia within 24 hours after a procedure is likely to indicate some degree of postoperative uvular injury [9-11]. The extent of uvular injury can lead to uvulitis and, in more severe cases, postoperative uvular necrosis [12,13]. Cases of postoperative uvular necrosis have been documented as early as 1978 following endotracheal intubation [11,14-16], upper gastrointestinal endoscopy [3,5,11], bronchoscopy via nasal approach [2,4], and in cases of vigorous suctioning [16]. Suction-induced pressure or trauma from oropharyngeal instrumentation impingement of the uvula's blood supply is believed to be responsible for the underlying pathophysiology of uvular necrosis [12-14].

A literature review by Reid et al. describes a higher prevalence of cases in the male population and patients younger than 40 years of age [15]. The difference in neck soft tissue and fat distribution between men and women has been previously studied and demonstrated to be of significant difference [16]. In males, increased neck soft tissue and fat distribution could explain the higher prevalence of uvular trauma [15,16]. Under anesthesia, the neck's soft tissue becomes flaccid and is more susceptible to mechanical injury [8,9].

The pediatric population forms a minority of reported cases regarding uvular injury, with a decreased tendency for progression to uvular necrosis and a higher prevalence of uvulitis [17]. In this demographic, symptom progression seems to occur more rapidly than in the adult population, typically occurring immediately after extubation [11,18]. An assessment of risk factors, such as procedure length, use of suction, type of device used, and the size of the endotracheal tube, revealed no identifying common factor except for the use of oropharyngeal instrumentation [15].

Postoperative uvular necrosis is a clinical diagnosis based on the examination of the uvula following a surgical procedure involving endotracheal access [15]. Visible changes and symptomatology may not be immediately apparent postoperatively, with an average of patients reporting symptoms after 24 hours [2,9,14,15]. As the necrosis worsens, patients may suffer from sore throats, dysphagia, globus sensations, odynophagia, dyspnea, and gagging [8,11,15]. On physical examination, the uvula is characterized by an elongated whitish tip with a black border, differentiating healthy tissue from necrotic tissue [9]. Evidence of necrotic discharge on the uvular surface can also be seen [8,9].

Current literature reports that patients with postoperative uvular necrosis typically have full resolution of symptoms within 14 days post procedure [2,8,15]. Nevertheless, our patient’s case highlights that the estimated 14-day recovery time for this postoperative complication is more likely based on symptom reporting at standard post-procedural follow-up appointments rather than on the actual day of symptom resolution. Due to its low incidence and prevalence, a widely accepted treatment regimen for postoperative uvular necrosis is yet to be developed [15]. Current options include supportive care with over-the-counter analgesics, single-dose corticosteroids, antibiotics, antihistamines, and surgical excision of necrotic portions [6,15]. Due to potential delays in wound healing secondary to steroid use, our patient was managed with supportive care and self-monitoring for the resolution of symptoms. Other common contraindications for the use of steroids include hypersensitivity reactions and active fungal infections [15]. Possible preventative techniques for the development of postoperative uvular necrosis include placing oropharyngeal devices away from the midline, avoiding blind suctioning, and decreasing the power of suctioning devices [2,6,15].

Due to the overall rarity of postoperative uvular necrosis, disease etiology and progression remain elusive. Optimal management, associated risk factors, and long-term complications continue to be areas of research partly due to a lack of reported data. Moreover, a major limitation of our case report is the lack of generalizability to the general population as a result of limited case reporting and varying levels of presentation. Being one of the few cases reporting the daily progression of symptoms and the absence of long-term complications, our case report lacks extensive comparison to the current literature.

## Conclusions

The importance of this case report ultimately lies in the detailed day-to-day description of symptom progression and subsequent resolution. This daily staging may provide patients and physicians with a potential expected postoperative course, as well as further guidance on the management of postoperative uvular necrosis. In addition, this case report is one of the first to provide information on any long-term sequelae of postoperative uvular necrosis. Subsequent cases should encourage patients to maintain a daily log of symptoms and self-imaging to help establish a more accurate average length of time between postoperative complication onset and ultimate resolution. Continuous postoperative follow-up would assist in confirming the absence of long-term sequelae described previously. Future cases should also explore additional methods to prevent postoperative uvular necrosis, even in patients with seemingly normal oral anatomy.
